# Nitrogen Enables Superior Strength–Ductility Synergy in Ultra-Low Carbon Steel via Copious Interphase Precipitation and Grain Refinement

**DOI:** 10.3390/ma19030622

**Published:** 2026-02-06

**Authors:** Qing Zhu, Rui Cao, Shuai Xu, Junheng Gao, Haitao Zhao, Qingxiao Feng, Hualong Li, Yixin Shi, Honghui Wu, Chaolei Zhang, Yuhe Huang, Jun Lu, Shuize Wang, Xinping Mao

**Affiliations:** 1 Institute for Carbon Neutrality, University of Science and Technology Beijing, Beijing 100083, China; d202110609@xs.ustb.edu.cn (Q.Z.); d202310777@xs.ustb.edu.cn (R.C.); xushuai@nercast.com (S.X.);; 2 Special Steel Research Institute, Central Iron and Steel Research Institute Co., Ltd., Beijing 100081, China; 3 Institute for Steel Sustainable Technology, Liaoning Academy of Materials, Shenyang 110004, China; 4 Jiangsu (ShaGang) Iron and Steel Research Institute, Zhangjiagang 215625, China

**Keywords:** nitrogen, precipitation strengthening, grain refinement, ultra-low carbon steel, tensile properties

## Abstract

The increasing use of electric arc furnace (EAF) in steelmaking inevitably elevates nitrogen (N) levels, which are traditionally regarded as a detrimental element to the formability of ultra-low carbon (ULC) steels due to the formation of Lüders band. Here, we demonstrate that N could act as a beneficial microalloying element in strip casting ULC steels by promoting V(C, N) precipitation and grain refinement of ferrite. Thermodynamic calculations reveal that N significantly increases both the equilibrium volume fraction and equilibrium precipitation temperature of V(C, N), enabling copious interphase nanoprecipitation during ferrite transformation. Microstructural characterization confirms the enhanced formation of V(C, N) within interphase rows in the N-containing steels, leading to greater Zener pinning effect and smaller ferrite grain size (from 7.50 μm of 0N to 4.67 μm of 96 ppm N and 3.84 μm of 139 ppm N). As a result, owing to the enhanced nanoprecipitation and grain refinement, the N-containing ULC strip casting steels exhibit a superior strength–ductility synergy, with tensile strength increased from 666 MPa (0N) to 805 MPa (96 ppm N) and 825 MPa (139 ppm N), and a slight decrease in total elongation from 29.8% (0N) to 27.3% (96 ppm N) and 22.0% (139 ppm N). In addition, no Lüders plateau was observed in the tensile stress-strain curves as the extensive formation of V(C, N) consumed the N atoms in solid solution. These findings highlight that microalloying V in the steels produced by EAF can effectively leverage the high N content for achieving superior strength–ductility synergy.

## 1. Introduction

Ultra-low carbon (ULC) steels (carbon content less than 0.03 wt.% [1]) are widely used in the automotive industry due to their excellent formability, weldability, and cost-effectiveness. Nevertheless, their relatively low strength remains a long-standing obstacle to broader utilization. Numerous strategies have been proposed to enhance the strength of ULC steels, including solid-solution strengthening [2], dislocation strengthening [3,4], grain refinement strengthening [5,6,7], and precipitation strengthening via microalloying with V, Nb, or Ti [8,9]. However, the enhancement in strength is often accompanied by degradation in ductility, resulting in poor strength–ductility synergy. Furthermore, the conventional precipitation-strengthening approach, i.e., the introduction of nanoscale carbide precipitates, is largely limited in ULC steels because the ultra-low carbon content limits the extensive precipitation of nano-carbides.

The development of strip casting provides an energy-efficient route that significantly reduces CO_2_ emissions compared with conventional continuous casting and hot rolling procedures [10]. However, two intrinsic technical characteristics of strip casting make the production of steels with high strength, large ductility, and high formability particularly challenging. First, to prevent peritectic reactions and avoid steel leakage, the carbon content in strip-casting steels is typically kept below 0.03 wt.%, which significantly hinders the extensive nanoprecipitation of carbides. Second, the increasing use of electric arc furnace (EAF), which is highly compatible with strip casting, inevitably introduces elevated N levels (40–100 ppm) in liquid steel during the steel making process, which would trigger the formation of Lüders band during cold working, thereby deteriorating the formability of steels [11].

In this study, we propose a novel V microalloying strategy for strip-casting ULC steels with high N content to achieve a pronounced precipitation-strengthening effect and a grain-refinement-strengthening effect. By introducing different N contents, we demonstrate that N can significantly enhance interface nanoprecipitation of V(C, N) during austenite-to-ferrite transformation, leading to an increased density of nanoprecipitates and substantial grain refinement of ferrite. As a result, a ULC steel consisting of obviously refined single ferrite phase with copious nanoprecipitates is achieved, yielding an exceptional strength–ductility synergy. In addition, no Lüders plateau was observed in the stress-strain curves. This study provides new insights into the design of ULC steels with high strength and large ductility.

## 2. Materials and Methods

**Figure 1:**
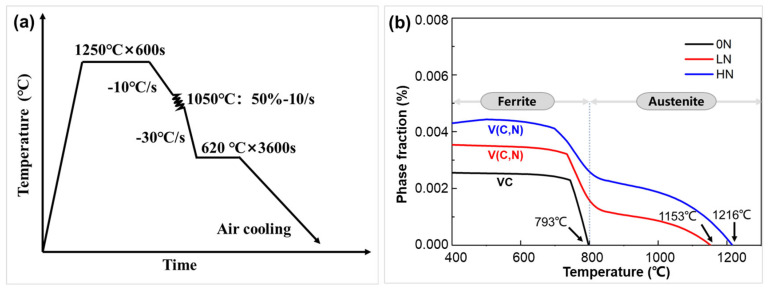
(**a**) Thermomechanical processing schedule of the 0N, LN, and HN steels and (**b**) calculated volume fractions of VC in the 0N steel and V(C, N) in the LN and HN steels as a function of temperature (Calculated using Thermo-Calc software, TCFE 12 database).

Three ULC steels with the same carbon content (0.025 wt.%) and vanadium content (0.2 wt.%), but different N levels, i.e.,0 ppm, 96 ppm, and 139 ppm, denoted as 0N, LN, and HN, were developed. The chemical compositions in Table 1 were measured values, with carbon and nitrogen determined by a carbon-sulfur analyzer (EMIA-920V2, HORIBA, Kyoto, Japan) and an oxygen-nitrogen-hydrogen analyzer (ONH836, LECO Corporation, St. Joseph, MI, USA), respectively, and other alloying elements by spark optical emission spectroscopy (SparkCCD 7000, NCS Testing Technology Co., Ltd., Beijing, China). The three steels were produced by vacuum arc melting followed by suction casting into a 5 mm-thick water-cooled Cu mold. The specimens were reheated to 1250 °C for 600 s, hot- rolled in a single pass at 1050 °C with 50% reduction, water-cooled to 620 °C, held for 60 min to simulate coiling, and then air-cooled to room temperature (Figure 1a). The thermomechanical processing route adopted here was designed to reproduce the key thermal-mechanical characteristics of industrial strip casting and has been used in prior studies for simulation of strip-casting-based processing [12]. Uniaxial tensile tests were carried out at room temperature using a universal testing machine (MTS Exceed 40, MTS Systems, Eden Prairie, MN, USA) at a strain rate of 6.67 × 10^−4^ s^−1^. To ensure the repeatability and accuracy of the mechanical properties, at least three independent tensile specimens were tested for each condition. Microstructural characterization was performed using electron backscatter diffraction (EBSD, Symmetry S2 detector, Oxford Instruments NanoAnalysis, High Wycombe, UK). Additionally, nanoprecipitation behavior was examined using transmission electron microscopy (TEM, Tecnai F30, FEI Company/Thermo Fisher Scientific, Waltham, MA, USA). The elemental composition of the nanoprecipitates was analyzed using energy-dispersive X-ray spectroscopy (EDS, Xplore, Oxford Instruments NanoAnalysis, High Wycombe, UK) integrated with the TEM. EBSD samples were prepared by electropolishing in 10% perchloric acid-alcohol at 20 V for 15 s. TEM foils were ground to 50 μm in thickness, punched into 3 mm discs, and electropolished in 5% perchloric acid-alcohol solution at −20 °C.

Thermodynamic calculations were performed using the Thermo-Calc software (version 2023a) in conjunction with the TCFE 12 (Thermo-Calc Fe-alloys) thermodynamic database. The equilibrium phase fractions of VC in the 0N steel and V(C, N) in the LN and HN steels were calculated based on the chemical compositions listed in Table 1. The calculations were conducted in the temperature range of 400 °C to 1300 °C under equilibrium conditions, focusing on the stability of austenite, ferrite, and the M (C, N) phase.

## 3. Results and Discussion

The thermodynamic calculation results of Figure 1b show the effect of N contents on the equilibrium phase fraction of VC in the 0N steel, and V(C, N) in the LN and HN steels. The results demonstrate that an increase of N content markedly elevates the formation temperature of V(C, N) from 793 °C (VC) in the 0N steel to 1153 °C in the LN steel and 1215 °C in the HN steel, while the equilibrium volume fraction of V(C, N) increases from 0.26% (VC) in the 0N steel to 0.36% in the LN steel and 0.43% in the HN steel. These results indicate that an increase in N content can significantly enhance the thermodynamic driving force for V(C, N) precipitation. Additionally, it is worth noting that all three steels (0N, LN, and HN) exhibit a sharp increase in equilibrium volume fraction of V(C, N) around 800 °C, the temperature at which the equilibrium austenite to ferrite transformation starts, reflecting a large solubility gap of V(C, N) between austenite and ferrite. It could be inferred that during the austenite to ferrite transformation, the sharp reduction in solubility provides thermodynamically favorable conditions for interphase precipitation [13,14]. However, owing to the rapid cooling (30 °C/s) after the single-pass rolling at 1050 °C (Figure 1a), ferrite transformation and associated V(C, N) precipitation are largely suppressed. Instead, these processes predominantly occur during the subsequent coiling stage, wherein the steel is held at a proper temperature of 620 °C—a favorable temperature for ferrite transformation and interphase nanoprecipitation.

**Figure 2:**
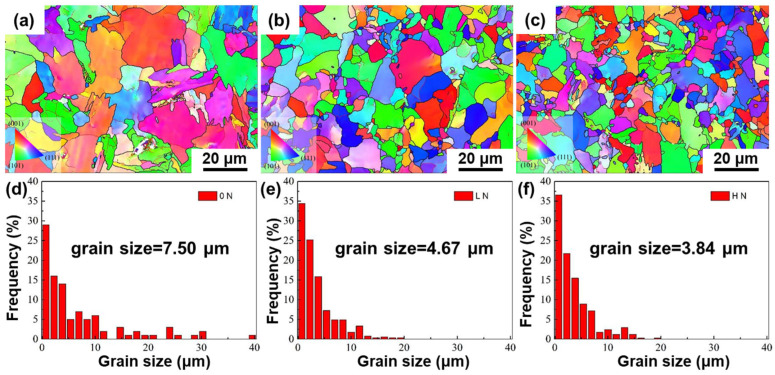
(**a**–**c**) EBSD inverse pole figure (IPF) maps and (**d**–**f**) their corresponding grain size distributions of the 0N, LN, and HN steels, respectively.

The microstructure of the 0N, LN, and HN steels was examined by EBSD (Figure 2). All three steels exhibit a fully polygonal ferrite microstructure, as shown in Figure 2a–c, demonstrating that the combined effects of ultra-low carbon content, high cooling rate after single-pass hot rolling, and appropriate coiling temperature effectively suppressed the formation of pearlite and bainite. However, as shown in Figure 2d–f, ferrite grain size shows a pronounced dependence on N content. The average ferrite grain size decreased markedly from 7.50 μm in the 0N steel to 4.67 μm in the LN steel and further to 3.84 μm in the HN steel, indicating that the increase of N content results in greater grain refinement effect.

**Figure 3:**
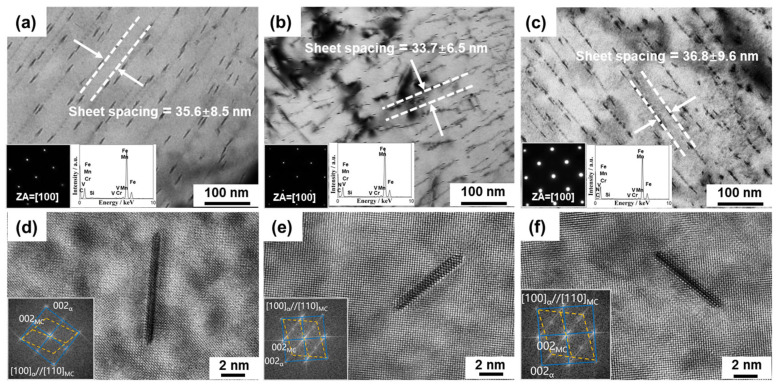
TEM characterization of nanoprecipitates in the (**a**,**d**) 0N, (**b**,**e**) LN, and (**c**,**f**) HN steels. (**a**–**c**) bright-field TEM images, and (**d**–**f**) HRTEM images with corresponding indexed FFT patterns (insets). The insets in (**a**–**c**) show the corresponding selected-area electron diffraction (SAED) patterns and TEM–EDS spectra.

TEM analysis was conducted to reveal the nanoprecipitation behavior of the 0N, LN, and HN steels (Figure 3). Bright-field images in Figure 3a–c demonstrate evident parallel interphase nanoprecipitation occurred in all three steels during austenite-to-ferrite transformation. TEM–EDS analysis (Insets in Figure 3a–c) shows V and C enrichment in the nanoprecipitates of the 0N steel, whereas an additional N signal is observed in the nanoprecipitates of the LN and HN steels, suggesting the formations of VC in the 0N steel and V(C, N) in the LN and HN steels, consistent with the thermodynamic calculation results in Figure 1b. Quantitative measurements (Figure 3a–c) show that the average row spacing of interphase precipitates remains nearly constant (35.6–36.8 nm) in the three steels, whereas the intra-row particle spacing decreases evidently from 20.1 nm in the 0N steel to 15.1 nm in the LN steel and 12.8 nm in the HN steel. These results demonstrate that N content has a negligible influence on the parallel row spacing of interphase precipitates but can substantially increase the number density of nanoprecipitates within each row. High-resolution TEM observations (Figure 3d–f) confirm that nanoprecipitates in the three steels share a disc-shape, with 1–2 nm in thickness and 5–10 nm in diameter, and maintain a Baker-Nutting orientation relationship with the ferritic matrix, as shown by the indexed Fast Fourier Transform (FFT) patterns in the insets of Figure 3d–f [15]. Importantly, although the volume fraction of V(C, N) increases with N content, the size of individual nanoprecipitate in the 0N, LN and HN steels remains nearly constant, indicating that N promotes precipitation without inducing precipitate coarsening.

**Figure 4:**
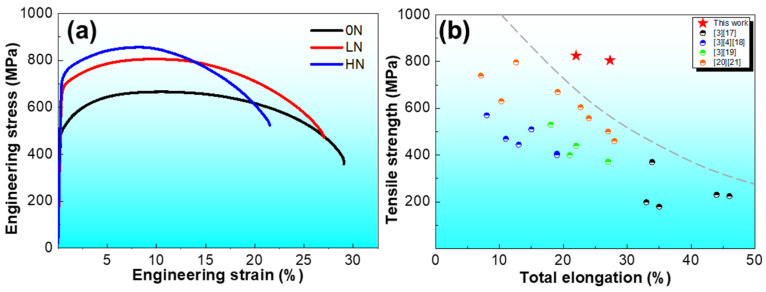
(**a**) Room-temperature tensile stress-strain curves of the 0N, LN, and HN steels and (**b**) comparison of ultimate tensile strength and total elongation with conventional ULC steels [3,17], dislocation strengthening ULC steels [3,4,18], grain refinement strengthening ULC steels [3,19], and precipitation strengthening ULC steels [20,21].

The tensile stress-strain curves of the 0N, LN, and HN steels are shown in Figure 4a. All three steels exhibit continuous yielding behavior without the observation of Lüders plateau, which is attributed to the fine ferrite microstructures and copious nanoprecipitation of V(C, N) that consumes almost all the N in the solid solution state [16]. Specifically, the 0N steel achieved a yield strength (YS) of 519 MPa, ultimate tensile strength (UTS) of 666 MPa, and total elongation of 29.8%. With N addition, the LN and HN steels exhibit significant increases in strength. The YS and UTS of the LN steel are 683 MPa and 805 MPa, respectively. With a further increase of N content, the YS and UTS of the HN steel reach 722 MPa and 825 MPa, respectively. Although the strength of the LN and HN steels increased significantly, in comparison with the 0N steel, total elongation of the two steels decreased slightly from 29.8% to 27.3% and 22.0%, respectively. Figure 4b shows the comparison of the strength and ductility of the LN and HN steels with other reported ULC steels, including conventional ULC steels [3,17], dislocation strengthening ULC steels [3,4,18], grain refinement strengthening ULC steels [3,19], and precipitation strengthening ULC steels [20,21]. Although diverse processing routes have been employed in the literature, Figure 4b suggests that the present V-N microalloying strategy can achieve a favorable strength–ductility synergy even under simplified strip casting simulation conditions. It can be concluded that in comparison with conventional ULC steels and ULC steels with specific strengthening mechanisms, e.g., grain refinement and nanoprecipitation, this V, N microalloying strategy makes the LN and HN steels exhibit high UTS (>800 MPa) and large elongation (>20%), demonstrating exceptional strength–ductility synergy.

The results in Figure 2a–c and Figure 3a–c collectively demonstrate that N markedly facilitates V(C, N) precipitation and promotes ferrite grain refinement in the LN and HN steels. Thermodynamic calculations in Figure 1b revealed that increasing N content markedly decreases the solubility product $K_{sp}$ of V(C, N), thereby leading to a greater extent of supersaturation. The corresponding chemical driving force for nucleation can be expressed as $∆G_{chem}=-RTln(Q/K_{sp})$, where Q denotes the activity product of dissolved solutes (V, C and N) in the matrix [22,23,24]. According to classical nucleation theory, the nucleation barrier decreases with increasing driving force ($∆G\propto 1/∆G_{chem}^{2}$), leading to a higher nucleation rate and thus a much greater number density of nanoprecipitates within each row in the LN and HN steels (Figure 3a–c). It should be noted that N has a relatively minor effect on the average parallel row spacing of interphase nanoprecipitates in the 0N, LN, and HN steels (Figure 3a–c). According to the classical ledge model of interphase precipitation [25], the parallel row spacing corresponds to the height of the ledge, which is mainly determined by the diffusion distance of alloying elements (e.g., V) at the interface. In this study, since the three steels have the same transformation temperature (620 °C) and alloying elements, their influence on the row spacing of interphase precipitation is negligible. Owing to the higher number density of nanoprecipitates within each row in the LN and HN steels, a greater Zener pinning force would be generated on ferrite grain boundaries [26], and thus cause higher grain refinement effect and smaller ferrite grain size in the LN and HN steels (Figure 2a–c).

The tensile testing results (Figure 4a) demonstrate that, in comparison with the 0N steel, the YS and UTS of the LN steel increased from 519 MPa and 666 MPa to 683 MPa and 805 MPa, and further increased to 722 MPa and 825 MPa for the HN steel, respectively. The strength increases in the LN and HN steels can be rationalized by the synergistic contributions of refined ferrite grains and copious formation of nanoprecipitates. Specifically, grain refinement provided an estimated ~80 MPa yield-strength increment calculated according to the Hall-Petch equation [27], while the reduced interparticle spacing from 20.1 nm to 12.8 nm yielded an additional ~80–120 MPa via enhanced precipitation strengthening, as calculated according to Ref. [12]. The high ductility of the LN and HN steels can be attributed to the following two aspects: (i) the significant refinement of ferrite grain size would promote more slip systems being activated during plastic deformation in comparison with that of their coarse-grain counterpart [28], facilitating more homogenous plastic deformation and alleviating stress concentration at grain boundaries, thus postponing microcrack nucleation and the occurrence of premature failure; (ii) the high density of nanoscale, regularly distributed nanoprecipitates in the LN and HN steels not only enhances the yield strength and tensile strength by the pronounced nanoprecipitation strengthening effect, but also contribute to maintaining the ductility by regulating dislocation behavior [29,30,31]. In particular, these regularly distributed nanoprecipitates promote the activation of additional slip systems and facilitate the uniform multiplication and dispersion of dislocations, thereby suppressing strain localization and contributing to the high ductility of the LN and HN steels. As a result, although the LN and HN steels exhibit tensile strengths exceeding 800 MPa, their elongations are as high as 27.3% and 22.0%, respectively, exceeding other ULC steels summarized in Figure 4b. This finding highlights that N, which is traditionally considered a tramp element detrimental to the formability of ULC steels, can be deliberately exploited as a beneficial alloying element to achieve superior strength–ductility synergy in strip casting ULC steels.

## 4. Conclusions

In summary, this study demonstrates that N plays a critical role in enabling copious V(C, N) precipitation in strip-casting ULC steels. Thermodynamic analysis established that N raises the thermostability and equilibrium volume fraction of V(C, N) at high temperature, thus thermodynamically facilitating the precipitation of V(C, N) during austenite to ferrite transformation, resulting in a higher number density and larger volume fraction of V(C, N) nanoprecipitates in the LN and HN steels. Microstructural observations confirmed that N promotes significant ferrite grain refinement due to the enhanced Zener pinning effect and the solute dragging effects of N atoms. The refinement of ferrite grain size and enhanced V(C, N) nanoprecipitation contribute to a remarkable enhancement in strength while maintaining adequate ductility, achieving an exceptional strength–ductility synergy in comparison with other ULC steels. The findings establish a new paradigm in alloy design for strip-casting steels, especially for steels produced by EAF with a characteristic of elevated N content.

## Figures and Tables

**Table 1:** Chemical compositions of the 0N, LN, and HN steels (wt.%).

	C	Si	Mn	Cr	V	N	Fe
0N	0.025	0.49	1.98	0.61	0.20	0	Bal.
LN	0.026	0.50	1.99	0.61	0.19	96 ppm	Bal.
HN	0.025	0.50	2.01	0.59	0.20	139 ppm	Bal.

## Data Availability

The original contributions presented in this study are included in the article. Further inquiries can be directed to the corresponding authors.
